# Role of secreted type I collagen derived from stromal cells in two breast cancer cell lines

**DOI:** 10.3892/ol.2014.2199

**Published:** 2014-05-30

**Authors:** SUNG HOON KIM, HYE YOON LEE, SEUNG PIL JUNG, SANGMIN KIM, JEONG EON LEE, SEOK JIN NAM, JEOUNG WON BAE

**Affiliations:** 1 Department of Surgery, The U Breast Surgery Center, Bundang-gu, Seongnam, Republic of Korea; 2 Department of Surgery, Division of Breast and Endocrine Surgery, Korea University Hospital, Korea University College of Medicine, Seoul 136-705, Republic of Korea; 3 Department of Surgery, Division of Breast and Endocrine Surgery, Samsung Medical Center, Sungkyunkwan University School of Medicine, Seoul 136-710, Republic of Korea

**Keywords:** breast cancer, collagen type I, fibroblast, neoplasm, matrix metalloproteinases

## Abstract

Collagen is one of numerous components of the cellular microenvironment. To date, the association between the microenvironment and tumorigenesis of malignant breast cancer remains elusive. Therefore, the aim of the present study was to investigate the potential role of a secretory protein of stromal cells, type I collagen, in the development of the aggressive characteristics of breast cancer cells. MDA-MB231 and MCF7 breast cancer cell lines were maintained in cultured media of normal human dermal fibroblasts (HDFs) and type I collagen-containing media. The morphological changes, adhesion capacity and matrix metalloproteinase (MMP)-1, -2 and -9 mRNA levels were evaluated. The results revealed that cell sprouting and adhesion capacity were enhanced in the MCF7 and MDA-MB231 breast cancer cells in HDF-conditioned culture media as well as in response to type I collagen treatment. The expression of MMP-9 mRNA was high in breast cancer cells cultured with the media of normal HDFs, compared with that of the control media. These data indicate that type I collagen, which is secreted by stromal fibroblasts, may augment the aggressive characteristics of breast cancer cells through the induction of MMP-9 mRNA.

## Introduction

The extracellular matrix (ECM) triggers numerous intercellular signaling pathways that regulate cellular growth, division, migration and differentiation (1). The ECM consists of numerous large macromolecules, including collagen, fibronectin and laminin (2). In the breast, collagens and other extracellular molecules may directly affect breast epithelial proliferation and differentiation (3). Type I collagen, the major structural component of the ECM, is a heterotrimer composed of three α-chains encoded by the collagen, type I, α1 (COL1A1) and COL1A2 genes (4). Type I collagen comprises ~84% of all the collagen synthesized by fibroblasts and large depositions of type I collagen lead to internal organ fibrosis (5). In particular, type I collagen acts as a physical barrier for cell migration and interferes with the proliferative ability of both normal and cancer cells (6).

Matrix metalloproteinases (MMPs) are a family of 24 human zinc binding endopeptidases which degrade components of the ECM and are associated with remodeling of the ECM and basement membrane under physiological conditions (7). Excessive expression of MMPs has been associated with numerous malignant processes, including cancer proliferation, invasion and metastasis, as well as inflammatory conditions, such as rheumatoid arthritis and osteoarthritis (8,9). Elevated plasma levels of soluble gelatinases, including MMP-2 and -9, have been positively correlated with a higher incidence of metastases in different types of cancer and have been considered as a valuable prognostic factor in breast and colon cancer (10). The degradation of type I collagen by MMP-1 and -9 has been associated with rapid progression, poor overall survival and secondary metastasis (11), and it appears this process may have a pivotal role in the acquisition of invasive characteristics in breast cancer (12,13). However, despite the numerous studies that have been conducted to further elucidate the cross-talking between malignant epithelial cells and stromal cells (14–16), the role of type I collagen, derived from fibroblasts, on the changing characteristics of breast cancer cells, has not been fully clarified. The present study examined the effect of type I collagen and culture media of normal human dermal fibroblasts (HDFs) on breast cancer cell lines.

## Materials and methods

### Cell lines and cell cultures

The human breast cancer cell lines, MCF-7 (luminal type A) and MDA-MB231 (triple negative, basal type) were cultured in Dulbecco’s modified Eagle’s medium (DMEM) supplemented with 10% fetal bovine serum (FBS), 100 IU/ml penicillin and 100 mg/ml streptomycin. Primary HDF cultures were obtained from the foreskin of healthy volunteers with their consent, aged 20–30 years old. The HDF cultures was provided by Dr Chung Jin Ho of Seoul National University (Seoul, Korea) (17). The isolated cells were allowed to attach to plastic plates and were cultured in DMEM supplemented with 10% FBS, 2 mM glutamine, 100 IU/ml penicillin and 100 μg/ml streptomycin. Following six or eight passages, the fibroblasts were used for the experiments. Each cell was maintained in a culture media supplemented without fetal bovine serum (FBS) for 16–24 h. DMEM, antibiotics (penicillin and streptomycin) and FBS were purchased from Life Technologies (Rockville, MD, USA). MCF-7 and MDA-MB231 were obtained from American Type Culture Collection (Manassas, VA, USA).

### Cell morphology study

MDA-MB231 and MCF7 breast cancer cell lines were seeded in the culture media of normal HDFs for 3 h and then analyzed. Each cell line was also seeded in serum free-DMEM media containing 50 μg/ml type I collagen (R&D Systems, Minneapolis, MN, USA) and water, respectively. The morphology of each cell was analyzed using the CK40 inverted microscope (Olympus, Tokyo, Japan).

### Dot-blotting

To verify the presence of secreted type I collagen in cultured media, MDA-MB231 and MCF7 cells and normal HDFs were incubated in serum-free media for 24 h. The indicated dose samples were transferred onto nitrocellulose membranes (Sigma-Aldrich, St. Louis, MO, USA) using the BIO-DOT™ apparatus (Bio-Rad, Hercules, CA, USA) and the membranes were then blocked with 10% skimmed milk in Tris-buffered saline with 0.01% Tween-20 (TBST; Sigma-Aldrich) for 15 min. The blots were incubated with mouse anti-human type I procollagen monoclonal antibody (SP1.D8; Developmental Studies Hybridoma Bank, Iowa City, IA, USA; dilution, 1:10) in 1% TBST buffer at 4°C overnight. Blots were washed three times in TBST and subsequently incubated in rabbit anti-mouse peroxidase-conjugated monoclonal antibody (Santa Cruz Biotechnology, Inc., Santa Cruz, CA, USA; dilution, 1:2,000) in TBST buffer. Following 1 h of incubation at room temperature, the blots were washed three times in TBST and enhanced chemiluminescent reagents (Amersham Biosciences, Buckinghamshire, UK) were used for development.

### Quantitation of type I collagen and MMP-9 mRNA

Total RNA was extracted from cells using TRIzol reagent (Invitrogen Life Technologies, Carlsbad, CA, USA), according to the manufacturer’s instructions. Isolated RNA samples were then used for reverse transcription polymerase chain reaction (RT-PCR). The samples (total RNA, 1 μg) were reverse transcribed into cDNA in 20 μl reaction volume using a first-strand cDNA synthesis kit for RT-PCR, according to the manufacturer’s instructions (MBI Fermentas, Hanover, MD, USA).

Gene expression was quantified by quantitative PCR (qPCR) using SensiMix SYBR kit (Bioline Ltd., London, UK) and 100 ng of cDNA/reaction. The sequences of the primer sets used for this analysis were as follows: human MMP-1: Forward, 5′-ATT CTA CTG ATA TCG GGG CTT TGA-3′ and reverse, 5′-ATG TCC TTG GGG TAT CCG TGT AG -3′; human MMP-9: Forward, 5′-CCC GGA CCA AGG ATA CAG-3′ and reverse, 5′-GGC TTT CTC TCG GTA CTG-3′; and human GAPDH (as an internal control): Forward, 5′-ATT GTT GCC ATC AAT GAC CC-3′ and reverse, 5′-AGT AGA GGC AGG GAT GAT GT-3′. An annealing temperature of 60°C was used for all of the primers. PCR was performed in a standard 384-well plate format with an ABI 7900HT qPCR detection system (Applied Biosystems, Foster City, CA, USA). For data analysis, the raw threshold cycle (CT) value was first normalized to the housekeeping gene for each sample to obtain the change in CT (ΔCT). The normalized ΔCT was then calibrated to control cell samples to obtain the ΔΔCT.

### Cell adhesion assay

MDA-MB231 and MCF7 breast cancer cells were seeded with serum-free media and 50 μg/ml type I collagen-containing media for 3 h on a 96-well plate. MDA-MB231 and MCF7 breast cancer cells were seeded with the culture media of breast cancer cells or normal HDF for 3 h on a 96-well plate.

To analyze the adhesion capacities of each cell, the cells were incubated with 5 mg/ml of 3-(4,5-dimethylthiazol-2-yl)-2,5-diphenyltetrazolium bromide solution (Sigma-Aldrich) at 37°C for 1 h. The culture media was removed and then the cells were dissolved with dimethylsulfoxide (Sigma-Aldrich). The adhesion capacity of each cell was analyzed at a wavelength of 595 nm on a spectrophotometer (Spectra Max 190; Molecular Devices, Sunnyvale, CA, USA).

### Statistical analysis

Student’s t-test was used to compare the cell adhesion rates with the mRNA expression levels. Statistical analyses were performed using PASW^®^ Statistics 18.0 (SPSS, Inc., Chicago, IL, USA). The results are presented as the mean ± standard error of mean. All P-values were two-tailed and P<0.05 was considered to indicate a statistically significant difference.

## Results

### Expression of type I collagen protein and mRNA in breast cancer cells and normal HDFs

**Figure 1:**
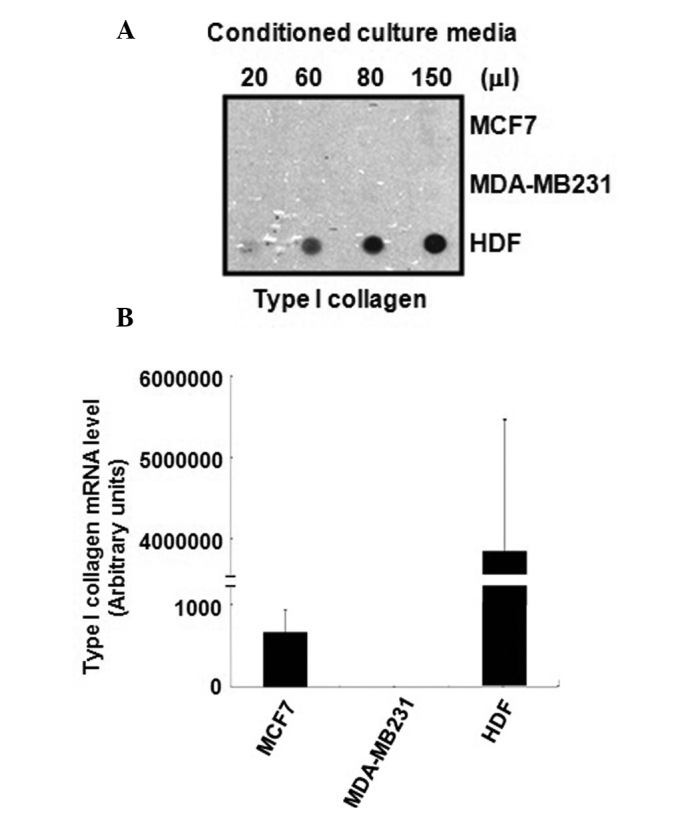
Expression of type I collagen secreted by each type of cell. (A) Type I collagen is not expressed in MCF7 and MDA-MB231 cells, but is expressed in HDF culture media, volume-dependently. (B) By quantitative polymerase chain reaction analysis, type I collagen is observed to be highly expressed, but only in HDF-conditioned culture media. HDF, human dermal fibroblasts.

The level of type I collagen protein expression in the conditioned culture media of MCF7 or MDA-MB231 breast cancer cells and normal HDFs was observed in this study. As demonstrated in Fig. 1A, type I collagen was not detected in the culture media of MCF7 and MDA-MB231 breast cancer cells, and was only detected in the culture media of normal HDFs. To verify the non-specific binding of type I collagen antibodies, type I collagen was loaded at the indicated doses in each well. It was revealed that the level of type I collagen protein expression increased in a dose-dependent manner (Fig. 1A). The expression of type I collagen mRNA was high in the HDF-conditioned culture media, whereas it was low or absent in the culture media of the breast cancer cell lines (Fig. 1B).

### Morphological changes of human breast cancer cells in HDF-conditioned cultured media

**Figure 2:**
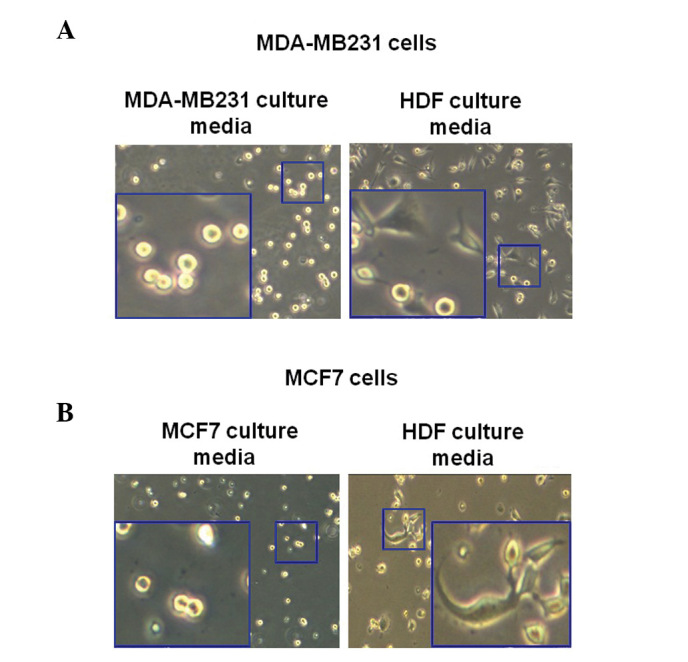
Morphology of human breast cancer cells in the HDF-conditioned culture media. (A) MDA-MB231 cells exhibited sprouting in the HDF-conditioned culture media, but no sprouting was evident in the MDA-MB231 culture media. (B) MCF7 cells exhibited sprouting in the HDF-conditioned culture media, but no sprouting was observed in the MCF7 culture media. HDF, human dermal fibroblasts.

To observe the change of morphology of breast cancer cells as induced by HDF-conditioned culture media, MDA-MB231 cells were incubated with or without culture media of normal HDFs for 3 h. In the present study, MDA-MB231 and MCF7 cells were trypsinized and then seeded with the culture media of each cell. Following 3 h, the morphology of cells was observed using a CK40 inverted microscope. As illustrated in Fig. 2A, the MDA-MB231 breast cancer cells exhibited enhanced sprouting in the HDF-conditioned culture media (Fig. 2A). However, the morphology of the MDA-MB231 breast cancer cells in the culture media of MDA-MB231 cells did not change (Fig. 2A). As revealed in Fig. 2B, the morphology of MCF7 cells markedly altered in the normal HDF culture media.

### Adhesion capacity of human breast cancer cells in HDF-conditioned culture media

**Figure 3:**
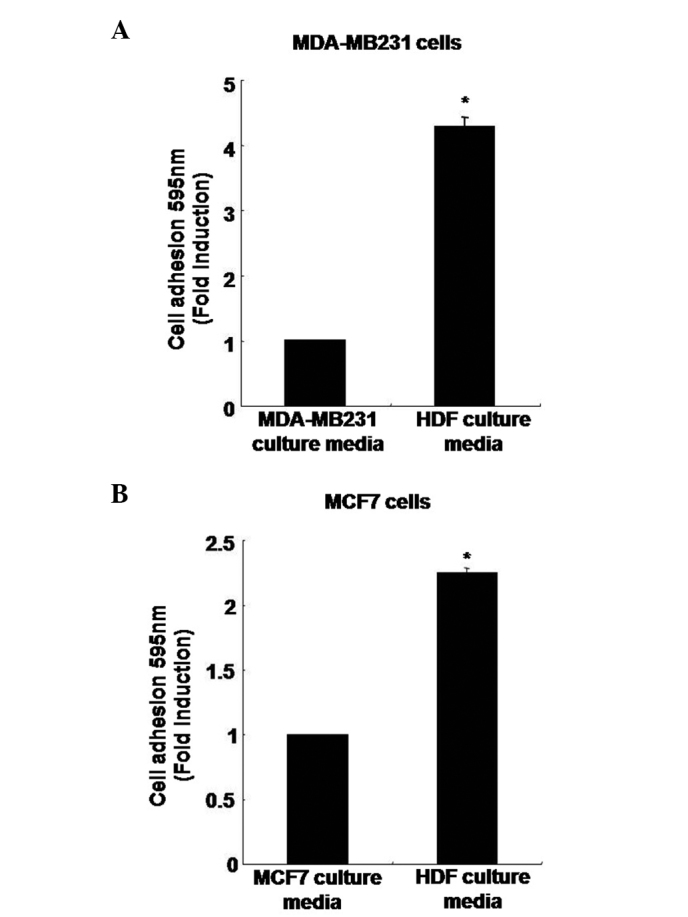
Adhesion rate of human breast cancer cells in HDF-conditioned culture media. (A) The adhesion rate of MDA-MB231 cells in the HDF-conditioned culture media significantly increased compared with that in the MDA-MB231 culture media. (B) The adhesion rate of MCF7 cells significantly increased in the HDF-conditioned culture media compared with that in the in MCF7 culture media. HDF, human dermal fibroblast.

To analyze the rate of adhesion capacity, MDA-MB231 cells were treated with the MDA-MB231 breast cancer cell culture media or the culture media containing normal HDFs. After 3 h, the adhesion capacity of MDA-MB231 breast cancer cells had increased by 4.27±0.15-fold when cultured in the HDF-conditioned culture media compared with the MDA-MB231 culture media (Fig. 3A). In addition, the adhesion capacity of the MCF7 breast cancer cells was also significantly higher, with an increase of 2.25±0.03-fold in the HDF-conditioned culture media compared with the MCF7 culture media (Fig. 3B).

### Type I collagen-induced morphological changes in breast cancer cells

**Figure 4:**
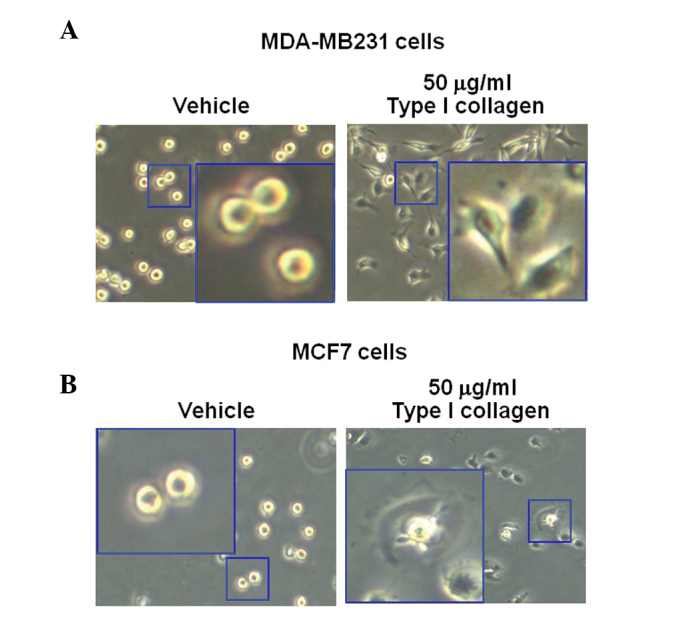
Morphology of breast cancer cells following treatment with type I collagen. (A) MDA-MB231 cells spread in type I collagen treated culture media, but not in the vehicle. (B) MCF7 cells also sprout in type I collagen treated culture media, but not in the vehicle.

MDA-MB231 and MCF7 breast cancer cells were treated with 50 mg/ml type I collagen. After 3 h, the morphology of the cells was observed using an CK40 inverted microscope. The results revealed that type I collagen augmented the sprouting of the cells in the MDA-MB231 and MCF7 breast cancer cell lines, but not in the vehicle-treated cells (Fig. 4A and B). These results were similar to those in Fig. 2.

### Type I collagen induces changes in the adhesion capacity of human breast cancer cells

**Figure 5:**
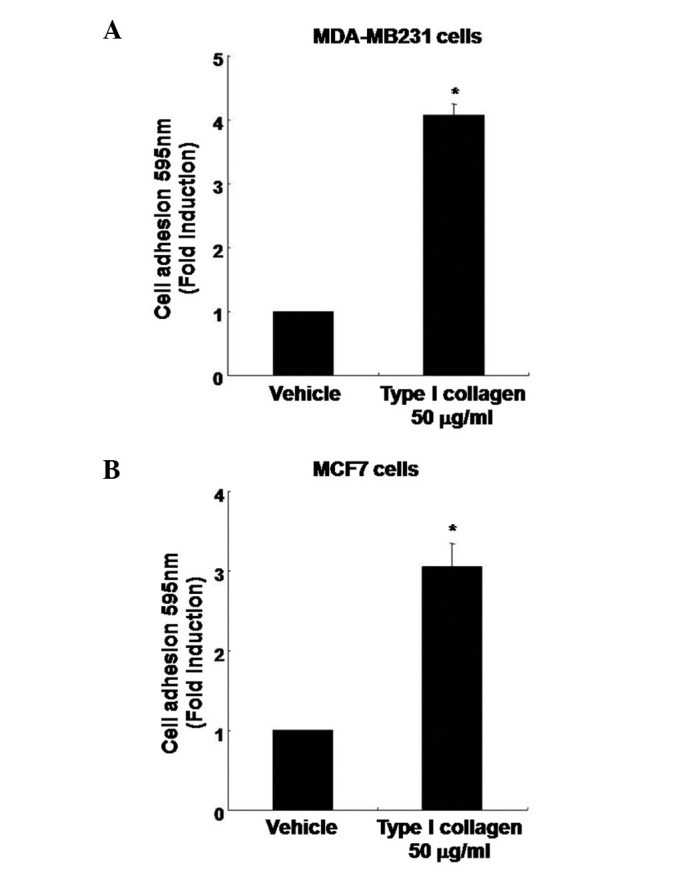
Adhesion rate of breast cancer cells following treatment with type I collagen. (A) Adhesion rate of MDA-MB231 cells significantly increased in type I collagen-treated culture media compared with the vehicle. (B) Adhesion rate of MCF7 cells also significantly increased in type I collagen-treated culture media compared with the vehicle.

To verify the effect of type I collagen on the adhesion capacity of MDA-MB231 cells by type I collagen, cells were treated with type I collagen at the indicated concentration for 3 h. Treatment with type I collagen enhanced the adhesion capacity of MDA-MB231 cells by 4.37±0.88-fold that of the vehicle-treated cells (Fig. 5A). The adhesion capacity of MCF7 breast cancer cells was also markedly increased by 3.72±0.52-fold (Fig. 5B).

### Expression of MMP-1, -2 and -9 mRNA in the culture media of breast cancer cells and normal HDFs

**Figure 6:**
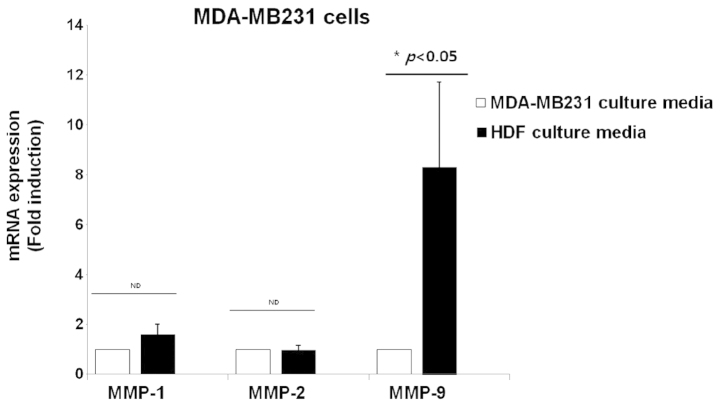
mRNA expression of MMP-1, -2 and -9 in the HDF-conditioned media. There was no significant difference in the expression levels of MMP-1 and -2 between the MDA-MB231 culture media and HDF-conditioned culture media. However, the expression of MMP-9 significantly increased in the HDF-conditioned culture media, compared with the MDA-MB231 culture media (P<0.05). MMP, matrix metalloproteinase; ND, no difference.

The effect of HDF-conditioned culture media on the expression of MMP-1, -2 and -9 was examined, which are all marker proteins of cancer metastasis. MDA-MB231 breast cancer cells were treated with culture media of MDA-MB231 and normal HDFs for 24 h, and cell lysates were harvested for detecting the level of MMP-1, -2 and -9 mRNA expression. It was demonstrated that the levels of MMP-1 and -2 mRNA expression were not affected by the normal HDF-conditioned culture media (Fig. 6). However, the level of MMP-9 mRNA expression was significantly increased by 8.3±3.4-fold in the HDF-conditioned culture media compared with the culture media of MDA-MB231 cells (Fig. 6).

## Discussion

ECM macromolecules are a major component of the cellular microenvironment and are in immediate contact with tumor cells (18). ECM molecules, including type I collagen, inhibit the proliferation of tumor cells by upregulating p27^KIP1^ in human M24met melanoma cells (19). In addition, type I collagen significantly augments the apoptotic cell death of MCF7 breast cancer cells through the existence of membrane type-1 (MT1)-MMP (8). In the present study, the correlation between the secreted proteins of normal fibroblasts and malignant breast cancer cells was investigated.

The interactions between malignant epithelial cells and their microenvironment are well established in tumorigenesis (20). The ECM is a key component of the microenvironment and affects numerous characteristics of tumor cells, including cell growth, survival and angiogenensis (18). The ECM molecules, including collagen, glycosaminoglycans and elastic fibers secreted from fibroblasts (which are a component of stromal cells), are key to its effects on tumorigenesis (5). These fibroblasts have an important role in the synthesis and remodeling of a variety of ECM molecules in the tumor stroma (21). In a previous study, Maquoi *et al* reported that collagen affects the fate of malignant epithelial cells in breast cancer cells through MT1-MMP-dependent mechanisms (8). In accordance with these investigations, the present study identified that two breast cancer cell lines, MDA-MB231 and MCF7, exhibited increased sprouting and enhanced adhesion rates following treatment with HDF-conditioned culture media. Therefore, it was demonstrated that secreted proteins of fibroblasts may affect the characteristics of breast cancer cells, which is consistent with the results of several other studies (21–23).

It is well established that carcinoma-associated fibroblasts stimulate cancer cell progression, through the secretion of various cytokines, such as stromal cell-derived factor-1 and transforming growth factor β (23,24). In the present study, however, another stimulatory pathway of tumor cells was investigated. MMPs contribute to a variety of malignant processes, including tumor growth, invasion and metastasis (9). In numerous types of cancer, elevated plasma levels of soluble gelatinases, such as MMP-2 and -9, have been positively correlated with a higher incidence of MMP-9 expression, and appear to be regulated by the binding of multiple factors, including nuclear factor κ-light-chain-enhancer of activated B cells and the activator protein 1, to their response elements (13,25). Kim *et al* reported that the basal level of MMP-9 expression was significantly increased by constitutively active mitogen-activated protein kinase kinase overexpression in SKBR3 breast cancer cells (13). Therefore, MDA-MB231 cells were treated with MDA-MB231 culture media and HDF-conditioned culture media, and MMP-1, -2 and -9 expression was analyzed. As a result, there was no difference in the expression of MMP-1 and -2 between the two media; however MMP-9 was highly expressed in the HDF-conditioned culture media with MDA-MB231 cells. Based on these results, it was assumed that fibroblasts may stimulate breast cancer cell metastasis and MMP-9 may effect this process. As mentioned above, MMP-9 is known to degrade basement membranes in breast cancer, and several studies have also reported that MMP-9 may be associated with breast cancer initiation and progression through interactions between oncogenes and tumor suppressor genes (12,26).

In a recent study, one of the microenvironment components, type I collagen, induced apoptotic cell death in luminal-like breast carcinoma cells but not in basal-like breast carcinoma cells (8). Secreted proteins of the microenvironment, including type I collagen and laminin, are important in the invasiveness and progression of breast cancer cells (27). The results of the present study reveal that type I collagen may be one of the ECM proteins which stimulates breast cancer cell metastasis, which is a result that is consistent with other studies (28–30). It is evident that type I collagen is not the only factor that initiates and promotes cancer cells to become metastatic, among other products of fibroblasts. However, it may be assumed that type I collagen has an important role in the development and initiation of metastasis in breast cancer cells. Further elucidating the detailed mechanisms underlying the effect of type I collagen on breast cancer cells requires further study. One investigation demonstrated that females with highly dense breasts, which is associated with a high density of type I collagen, also had a higher risk of recurrence following a mastectomy or radiotherapy (31). Therefore, it is possible that type I collagen may be considered as a potential therapeutic target or prognosis factor of the disease.

In the present study, the results revealed that HDF-conditioned culture media augmented the aggressiveness of breast cancer cells, through the induction of sprouting cells and the enhancement of adhesion capacities. In addition, being one of the key enzymes of metastasis, the expression of MMP-9 mRNA was significantly enhanced by the HDF-conditioned culture media. Based on this evidence, the present study demonstrates that microenvironmental sources, including secreted cytokines and proteins (i.e. type I collagen) of stromal fibroblasts cells, may induce the development of aggressive characteristics in breast cancer cells.
